# Notes on Some of the Newer Remedies Used in Diseases of the Skin

**Published:** 1897-03

**Authors:** 


### Notes on Some of the Newer Remedies Used in Diseases of the Skin.

We are in receipt of a reprint, of "an Address of the Chairman, delivered in the Section on Dermatology and Syphilography, at the 47th annual meeting of the American Medical Association, held at Atlanta, Ga., May 5–6, 1896. By L. Duncan Bulkley, A. M., M. D., New York." Reprinted from the *Journal of the American Medical Association*, November 28, 1896. Dr. Bulkley gives his experience with the use of certain remedies in the New York Skin and Cancer Hospital, and in private practice.

The following extracts will be of interest to our readers:

"*Antipyrin*, while not a very new remedy for general use, has recently been advocated in urticaria and will often prove very effective, provided there is not some alimentary disorder continuing the trouble. When cases have resisted ordinary remedies, a moderate dose of antipyrin, given three or four times daily, between meals, will often serve to stop the tendency to the eruption. Phenacetin, and even antifebrin, will sometimes prove of great service as antipruritics, especially when given at night.—*Bulkley*.

"In syphilis, in the early stages, of all the numerous preparations of mercury recommended, there is no one to which I can refer with special recommendation, * * * and I have yet to find any yield better results (in early syphilis) than the one grain tablets of mercury and chalk, given every two hours, as recommended by Jonathan Hutchinson." [It will be remembered that Dr. Shoemaker, in his Clinical Lecture, in our last issue, recommended this treatment.—Ed.]

Dr. Bulkley says:

"Turning now to the local treatment of diseases of the skin, we find that a host of new remedies have been presented of late years; among these many have not fulfilled the expectations which were raised, while many are of very considerable value, and their worth has been confirmed by many observers.

"*Resorcin* stands prominent among these, and of its value, when properly used, there can be no doubt. To Unna is due the credit of pressing the importance of this remedy upon the profession, mainly in connection with the eczema seborrhoicum, with which his name has become inseparably connected. In this eruption, which in reality is no eczema, but a parasitic disease of microbic origin, resorcin is almost a sovereign remedy. In a strength of about 6 per cent. in zinc ointment, or in solution with a little alcohol and glycerin, it will often clear off a well-defined eruption in a very few days. The solution is particularly applicable in the scalp, and the surface should be thoroughly wet with it morning and night by means of a large medicine dropper; it will thus commonly arrest at once the itching attending the scaling of the scalp, which is often one of the earliest signs of seborrheic eczema, and which often leads to a loss of the hair.

"Resorcin, used much stronger, even up to 25 per cent., in zinc ointment, will sometimes give brilliant results, locally, in the treatment of acne rosacea. The application is kept on for several days, and causes some little inflammation, after which the previous redness and pustular condition will be found to have largely disappeared. A second or third application may be necessary, and if the cause of the reflex congestion has been removed by appropriate diet and medication, there will be very much permanent benefit. Resorcin also proves serviceable in certain ulcerative conditions, notably those of a tuberculous type, used in a mild ointment, not exceeding 10 per cent.

*Ichthyol* certainly stands next in importance among the newer additions to therapeutics in dermatology, as it is also valuable in other branches of medicine, and all are undoubtedly familiar with its use. As an antipruritic it is often of great service. Added to ointments, in a strength of from 6 to 10 per cent., it is very valuable in eczema, and may be used in even quite acute conditions. In dermatitis herpetiformis, a watery solution, 5, 10 or even 20 per cent. will often give more relief than any other local remedy. When the skin is too dry it can be used in almost the same strength in oil with much advantage; the same measures are of much service in pruritis ani. In burns an ichthyol ointment, about 6 per cent., will often prove the most comfortable dressing, and on old ulcers of the leg the same, though stronger, is very valuable.

"Ichthyol has a power of reducing infiltrations, and in chronic conditions may be painted pure over the surface with much advantage. I have a number of times seen the greatest benefit result from painting pure ichthyol over joints enlarged by rheumatism and gout, and then applying one or two thicknesses of flannel, wrapped firmly on the part, forming an adherent dressing with the ichthyol. This may be removed and fresh ichthyol painted on daily, and wrapped with the same flannel; patients who have long suffered from these conditions have obtained more relief from this method of treatment than from any previously adopted.

